# Anaesthesia as a risk factor for long-term cognitive decline: Results of the prospective MAAS cohort study

**DOI:** 10.1097/EJA.0000000000002133

**Published:** 2025-02-17

**Authors:** Christoph H. Pennings, Martin Van Boxtel, Dianne De Korte-De Boer, Wolfgang Buhre, Carine J. Vossen

**Affiliations:** From the Department of Anaesthesiology and Pain Medicine, Division for Acute and Critical Care, Maastricht University Medical Centre, The Netherlands (CHP, DDeK-DeB, CJV), School for Mental Health and Neuroscience, Maastricht University, The Netherlands (MVB), and Department of Anaesthesiology, Division of Vital Functions, University Medical Centre Utrecht, The Netherlands (WB)

## Abstract

**BACKGROUND:**

There are concerns whether (repeated) exposure to general anaesthesia is associated with long-term cognitive decline.

**OBJECTIVE:**

We investigated the potential, negative relationship between total exposure to surgery under general anaesthesia and its impact on long-term cognitive development.

**DESIGN:**

A prospective longitudinal cohort study.

**SETTING:**

The Netherlands.

**PARTICIPANTS:**

1823 Adults, aged 25–84 with normal cognitive functioning on inclusion with three serial cognitive assessments between 1995 and 2008, with comprehensive documentation on demographic, lifestyle, and health factors.

**MAIN OUTCOME MEASURES:**

The primary outcomes were test scores in the cognitive domains of learning and memory, executive function, selective attention, mental speed, and information processing speed. Linear mixed models were used to analyse the effects of the estimated total time under general anaesthesia at baseline on cognitive development during a 12-year follow-up period.

**RESULTS:**

When adjusting for demographic and systemic health-related factors, prolonged exposure to surgery under general anaesthesia (measured in total baseline minutes) negatively affected three cognitive domains. These included the CST (executive functioning, *P* < 0.05), Stroop (selective attention and mental speed, *P* < 0.001) and LDST (information processing speed, *P* < 0.005). Age and education were the primary factors impacting lifetime cognitive decline. Hypertension, diabetes, and smoking negatively affected various cognitive domains.

**CONCLUSION:**

Increased exposure to surgery under general anaesthesia independently contributes to long-term cognitive decline. Demographic variables and health-related factors are key contributors to accelerated cognitive decline over an individual's lifetime.

**KEY POINTS**

- There are concerns that exposure to general anaesthesia is associated with long-term cognitive decline.
- Little is known about the influence of prolonged exposure to anaesthesia on the natural, long-term continuum of cognitive decline.
- Our study indicates that prolonged exposure to surgery under general anaesthesia during an individual's lifetime significantly affects long-term cognitive decline.
- Cognitive trajectories appear to be primarily affected by demographic (age and educational level) and systemic health-related factors (hypertension, diabetes and hypercholesterolemia).

## Introduction

Cognitive development follows a natural continuum over the course of the human lifespan.^1^ In addition to age-related cognitive decline, several health-related factors, including hypertension, diabetes mellitus, and cardiovascular disease, contribute to the accelerated decline in cognitive function.^2–4^. Within the field of anaesthesiology, there are concerns regarding the possible effects of surgery and anaesthesia on hastening cognitive decline.^5,6^ These concerns are strengthened by the fact that the ageing population faces an increasing need for surgical interventions under general anaesthesia during their lifetime.^7^

Perioperative neurocognitive disorders (PND) are frequent complications after surgery and general anaesthesia, with an estimated incidence of 11.7% up to 63%.^2,3,8^ PNDs may occur preoperatively, immediately postoperatively (e.g., postoperative delirium), in the postoperative period (e.g., delayed neurocognitive recovery after surgery up to 30 days), or as a postoperative neurocognitive disorder up to 12 months and even extending beyond that period.^9^ Patients with PND have poorer functional recovery, prolonged hospital stay, and increased use of healthcare resources.^10,11^

Although transient cognitive decline after non-cardiac surgery and anaesthesia is an established complication,^12,13^ its long-term effects on cognitive functioning have not been well studied. One study, the International Study of Postoperative Cognitive Dysfunction (ISPOCD 1), indicated that short-term PND does not affect the long-term occurrence of dementia. In addition to the ISPOCD study and subsequent follow-up analysis after 11 years, limited long-term evidence is available regarding the effects of surgery under general anaesthesia on cognitive trajectories beyond 1 year.^14^ In 1998, a cross-sectional study examined the impact of lifetime anaesthetic exposure on age-related cognitive functioning. Increased exposure to general anaesthesia was not considered a factor for increased age-related cognitive decline.^15^

The present study is a sub-analysis of the Maastricht Aging Study (MAAS), which investigated patterns of cognitive ageing and the influence of multiple factors in the general population within a 12-year follow-up period.^17^ The MAAS study cohort provides a unique framework for investigating the impact of surgery under general anaesthesia in a well controlled setting, with an extensive follow-up period and registration of independent risk factors for cognitive decline. This study aimed to test the hypothesis that prolonged exposure to surgery under general anaesthesia leads to increased cognitive decline over a 12-year follow-up period.

## Methods

### Study design and participants

The MAAS study was initiated to investigate the determinants of cognitive aging, and its design and objectives have been outlined in a previous paper.^16^ MAAS is a prospective cohort study of individuals without psychiatric or neurological conditions that may directly impact cognitive function at baseline. Participants aged 24–86 were recruited from the Research Network Family Medicine Maastricht (RNFM). The RNFM is a patient registry of general practitioners managed by the Department of General Practice at Maastricht University. The patients in the registry are representative of the overall Dutch population. A total of 10 801 Individuals from the RNFM were invited to participate in the study. Exclusion criteria included chronic neurological pathology (e.g., dementia, Parkinson's disease, epilepsy), cerebrovascular disease, mental retardation, or psychotropic drug use. After checking for eligibility, 1823 participants were selected for baseline assessment using an optimal stratified sampling design with strata for age (13 levels, ranging from 25 ± 1 years to 80 ± 1 years), sex (male/female), and occupational achievement (low/high). The baseline examinations were conducted between 1993 and 1996. Participants underwent repeated assessments of medical status, lifestyle, and anthropomorphic and cognitive measures at baseline and six and 12 years.

### Ethics

All participants provided informed consent prior to participation. Ethical approval for this study (METC-05-107) was granted by the Maastricht University Medical Centre Ethics Committee (METC aZM/UM). After the original approval, several extensions were granted by the METC aZM/UM, the latest of which was chaired by Professor Dr C. de Bie-Smulders at P. Debyelaan 25, 6202 AZ, Maastricht, on 19 September 2009.

## Exposure

### Total time under general anaesthesia

Participants self-reported whether they had undergone surgery, the number of surgeries, and the types of surgeries they had during baseline assessment. Patients were then thoroughly interviewed to verify the details of the surgical procedures they underwent. Based on these patient-reported events, an experienced anaesthesiologist from Maastricht University Medical Centre estimated the typical duration of each procedure under normal circumstances. The procedures were categorized into four groups: less than 30 min, 30 min to 1 h, 1 to 3 h, and more than 3 h. For our analysis, we used the midpoint of each category to estimate the total time under general anaesthesia.

### Cognitive assessment

Psychologists or trained assistants performed neuropsychological tests at baseline and the 6- and 12-year follow-up. Four cognitive domains were assessed: verbal memory, selective attention and mental speed, executive functioning and information processing speed. The derivatives of these domains were used as outcome measures to model the cognitive trajectories.

The concept-shifting test (CST) assesses executive functioning.^17^ In three consecutive trials, participants crossed out digits as fast as possible in ascending order (Part A), letters in alphabetic order (Part B), and digits and letters in alternating order (Part C). The Shifting Score is a measure of executive function. The shifting score was calculated by subtracting the average time needed to finish parts A and B from the time required to complete part C and was used as the outcome measure.

The Visual Verbal Learning Test (VVLT) evaluates the verbal memory.^18^ The VLT was conducted by presenting 15 monosyllabic words in five trials on a computer screen, followed by immediate and delayed recall phases after 20 min. Delayed word recall was used to assess cognitive domains of verbal memory.

The Stroop Colour Word Test (SCWT) assesses mental speed, selective attention, and susceptibility to interference.^19^ The test involved three sheets of 40 stimuli each: colour names (Part 1), coloured patches (Part 2), and colour names printed in incongruously coloured ink (Part 3). The measured variable is the time in seconds needed to read either (Part 1), name the colour of the patches (Part 2), or the colour of the printed words (Part 3). An interference measure was calculated by subtracting the average time needed to complete the first two subtasks from the time needed to complete the third subtask (Interference = Stroop 3 – ((Stroop 1 + Stroop 2/ 2).

The letter digit substitution test (LDST) assesses an individual's information-processing speed.^20^ In this paper-and-pencil test, letters are matched with numbers according to a coding scheme as quickly as possible within 90s. The number of correct substitutions is included in the analysis.

### Covariates

Previous MAAS database research has identified several health-related conditions that affect cognitive function decline.^21^ We included several variables in the models: present or past smoking (yes/no); alcohol abuse, defined as consuming >14 units of alcohol per week for women, >21 units per week for men, or more than six units of alcohol on a single day per week, in accordance with Dutch health guidelines; type 2 diabetes (yes/no); hypertension (yes/no); hypercholesterolemia (yes/no); and history of coronary artery disease (including angina pectoris, myocardial infarction, bypass surgery, or open-heart surgery). The following potentially confounding demographic variables were also included: age, sex and educational level (categorized as low, middle or high)

### Statistical analysis

Baseline characteristics were reported for the same cohort at three time points: at baseline, after 6 years, and after 12 years of follow-up. All data were analysed using descriptive statistics. Categorical data are reported as frequencies and percentages. For continuous data, the means and standard deviations were calculated. All the parameters were checked for normality.^22^ The Stroop colour-word test and shifting score of the CST were negatively skewed and logarithmically transformed to an approximate normal distribution. Likewise, the delayed recall of the visual-verbal learning test required square transformation for optimal distribution.

Because of the hierarchical structure of our data, multilevel analysis was used to test the relationship between the total time under general anaesthesia and cognitive change. Each participant represented the highest level in our model, with repeated measurements of cognitive variables as the second level. We included a squared term for age in the model to account for the accelerated rate of cognitive decline with increasing age, as in previous MAAS studies.^23,24^ Additionally, an anaesthetic duration-by-follow-up time interaction term was included to model the effect of total time under general anaesthesia on cognitive decline over the follow-up period. Appendix A, Supplemental Digital Content describes the basic and full multilevel models used in the analysis.

First, the basic model examined the effect of total time under general anaesthesia and the interaction of total time under general anaesthesia with follow-up time, adjusted for demographic variables: age, age,^2^ sex and educational level. Next, the full model incorporated additional health-related covariates including smoking status, alcohol use, hypertension, type II diabetes, hypercholesterolemia, coronary artery disease, and demographic variables. The primary variable of interest was total time under general anaesthesia and its interaction with time.^21^ An AR-1 covariance structure was selected for repeated measures based on the lowest Bayesian criterion (BIC) and AIC values. An unstructured covariance structure was used for the random effects. All statistical analyses were performed using SPSS 28.0.1 (IBM Corp, Armonk, NY). R version 4.4.1, R Core Team. (2024).

## Results

The baseline and follow-up sample characteristics are summarised in Table 1. At baseline, there were 1823 participants, with seven cases (0.4%) of missing outcome data for the VLT, 23 cases (1.3%) for the Stroop, 32 cases (1.8%) for the CST and four cases (0.2%) for the LDST. At the 6-year follow-up, there were 1417 participants (77.8% of the baseline cohort), with 43 cases (3%) of missing data for the VLT, 62 cases (4.4%) for the Stroop, 66 cases (4.7%) for the CST and 46 cases (3.2%) for the LDST, based on the remaining sample. At the 12-year follow-up, there were 1188 participants (65.2% of the baseline cohort), with seven cases (0.6%) of missing data for the VLT, 16 cases (1.3%) for the Stroop, 18 cases (1.5%) for the CST, and five cases (0.4%) for the LDST, again calculated from the remaining sample.

**Table 1:** Cohort characteristics at baseline and follow-up

Variable	Baseline (*N* = 1823)	6-year follow-up (*N* = 1417)	12-year follow-up (*N* = 1188)
Age	51.1 (24–84)	56.01 (30–88)	59.66 (36–92)
Sex, male	913 (50.1%)	707 (51.3%)	590 (49.7%)
BMI	22.6 (3.7)	22.9 (3.74)	22.83 (3.95)
Education			
* Low*	665 (36.5%)	465 (33.8%)	311 (29.8%)
* Middle*	746 (41.0%)	588 (42.8%)	444 (27.6%)
* High*	410 (22.5%)	321 (23.4%)	288 (27.6%)
***Comorbidities***			
* *Smokers	525 (29.0%)	295 (22.4%)	189 (17.7%)
* *Hypertension	339 (18.6%)	296 (20.9%)	318 (26.8%)
* *Diabetes	86 (4.7%)	89 (6.3%)	106 (8.9%)
* *Coronary artery disease	171 (9.4%)	214 (15.1%)	201 (16.9%)
* *Hypercholesterolemia	173 (9.5%)	173 (8.1%)	177 (14.9%)
* *Alcohol abuse	125 (8.4)	108 (10.3%)	91 (10.6%)
***Anaesthesia***			
* *Number of anaesthesia (mean, SD)	1.78 (1.77)	2.10 (2,00)	2.25 (2,12)
* *Total anaesthetic time (min)	82.56 (0–840)	–	–
***Outcome measures***			
* *Visual verbal learning test delayed recall (verbal memory)^a^	10.12 (7.5)	11.16 (7,9)	11.26 (8,0)
* *Concept shifting test (executive function)^b^	30.0 (1.5)	30.0 (1,5)	33.1 (1,6)
* *Stroop colour word test (attention & mental speed)^c^	46.3 (23.4)	47.1 (27)	47.9 (34.6)
* *Letter digit substitution test (information processing speed)^d^	48.3 (11.8)	51.3 (13.0)	48.9 (13.3)

Data are *n* (%), mean ± SD. Age and total anaesthetic time are represented as mean (range). ^a^ Number of correct recalls. ^b^ Time in seconds. ^c^ Time in seconds. ^d^ Number of correct substitutions.

The mean number of surgeries performed under general anaesthesia was 1.78 (SD 1.77) at baseline, increasing to 2.25 (SD 2.12) after 12 years. Among the entire cohort, 463 participants (23.2%) did not undergo any surgery, 873 participants (47.9%) had undergone one to two surgeries, and 487 participants (26.7%) had undergone more than two surgeries under general anaesthesia at baseline. While participants reported the number of surgeries they underwent at each follow-up, total time under general anaesthesia was only estimated at baseline, with a mean of 82.56 min (SD 100.23 min, range 0 to 840). Data on total anaesthesia time at the 6- and 12-year follow-ups was not assessed.

A summary of the results of the basic and full models is presented in Tables 2 and 3. In the basic model, increased exposure to general anaesthesia at baseline was associated with a slight but significant decrease in performance in executive functioning (CST; Estimate 0.0002 (95% CI 0.0000 to 0.0003), *P* < 0.005), selective attention and mental speed (Stroop; Estimate 0.0002 (95%CI -0.015–0.02), *P* < 0.001), and information processing speed (LDST; −0.0038 (95% CI −0.006 to 0.002), *P* < 0.005) during the follow-up period. A persistent negative effect in all cognitive domains was associated with older age and a lower educational level.

**Table 2:** Summary of the basic model using linear mixed models. × indicates an interaction term

Cognitive test	Variable	Estimate	95% Confidence interval		*P* value
Concept shifting test	Intercept	3.1493	2.9970	3.3016	<0.001
*(executive functioning)*	Total anaesthetic time at baseline	−0.0001	−0.0002	0.0001	0.244
	Time (follow-up)	0.0835	0.0713	0.0956	<0.001
	Total anaesthesia time at baseline × time (minutes)	0.0002	0.0000	0.0003	<0,005
	Age at baseline	0.0068	0.0011	0.0125	0,019
	Age at baseline^2^	0.0001	0.0000	0.0001	<0.005
	Sex	−0.0049	−0.0315	0.0218	0.721
	Educational level	−0.1705	−0.1887	−0.1524	<0.001
Visual verbal learning test	Intercept	86.0351	62.5202	109.5499	<0.001
*(verbal memory)*	Total anaesthesia time at baseline	0.0145	−0.0087	0.0378	0.220
	Time (follow-up)	8.4070	6.5598	10.2542	0.000
	Total anaesthesia time at baseline × time (minutes)	−0.0058	−0.0216	0.0100	0.473
	Age at baseline	0.1075	−0.7673	0.9824	0.810
	Age at baseline^2^	−0.0167	−0.0251	−0.0082	<0.001
	Sex	22.6576	18.5473	26.7679	<0.001
	Educational level	14.4714	11.6781	17.2647	<0.001
					
Stroop colour word test	Intercept	3.9482	3.7782	4.1182	<0.001
*(attention & mental speed)*	Total anaesthesia time at baseline	−0.0003	−0.0005	−0.0002	<0,05
	Time (follow-up)	0.0210	0.0087	0.0334	<0.001
	Total anaesthesia time at baseline × time (minutes)	00003	0.0002	0.0004	<0.001
	Age at baseline	−0.0130	−0.0193	−0.0066	<0.001
	Age at baseline^2^	0.0003	0.0002	0.0003	<0.001
	Sex	−0.0689	−0.0987	−0.0391	<0.001
	Educational level	−0.1163	−0.1364	−0.0962	<0.001
Letter digit substitution test	Intercept	52.1930	47.8976	56.4884	<0.005
*(information processing speed)*	Total anaesthesia time at baseline	0.0038	−0.0002	0.0079	0.065
	Time (follow-up)	−0.5903	−0.8293	−0.3513	<0.005
	Total anaesthesia time at baseline × time (minutes)	−0.0038	−0.0059	−0.0018	<0.005
	Age at baseline	0.0324	−0.1277	0.1926	0.691
	Age at baseline^2^	−0.0049	−0.0064	−0.0033	<0.005
	Sex	0.8761	0.1199	1.6323	<0.05
	Educational level	4.0438	3.5442	4.5434	<0.005

CST: speed in seconds; higher scores mean slower task completion. VVLT: number of correct recalls; lower score means lower test performance. Stroop: time in seconds; a higher score is slower task completion. LDST: number of correct substitutions; a lower score means lower test performance.

**Table 3:** Full model using linear mixed model, × indicates an interaction term

Cognitive test	Variable	Estimate	95% Confidence interval		*P* value
Concept shifting test	Intercept	3.0426	2.8681	3.2172	<0.001
*(Executive functioning)*	Estimated total anaesthesia time at baseline	−0.0001	−0.0003	0.0011	0.1631
	Time (follow-up)	0.0818	0.0691	0.0946	<0.001
	Total anaesthesia time at baseline × time (minutes)	0.0001	0.0000	0.0002	<0.05
	Age at baseline	0.0081	0.0017	0.0146	<0.05
	Age at baseline^2^	0.0001	0.0000	0.0001	0.0577
	Sex	0.0122	−0.0179	0.0423	0.4258
	Educational level	−0.1675	−0.1874	−0.1477	<0.001
	Hypertension	0.0012	−0.0410	0.0434	0.9569
	Coronary artery disease	0.0311	−0.0280	0.0903	0.3023
	Diabetes Mellitus	0.1229	0.0422	0.2036	<0.005
	Hypercholesterolemia	0.0160	−0.0374	0.0694	<0.001
	History of smoking	0.0366	0.0044	0.0688	0.1631
	Problematic alcohol use	0.0145	−0.0390	0.0679	<0.001
Visual verbal learning test	Intercept	95.9562	68.6068	123.3056	<0.001
*(Verbal memory)*	Estimated total anaesthesia time at baseline	0.0134	−0.0130	0.0399	0.319
	Time (follow-up)	8.1137	6.1197	10.1077	<0.001
	Total anaesthesia time at baseline × time (minutes)	0.0027	−0.0150	0.0203	0.768
	Age at baseline	−0.3515	−1.3591	0.6561	0.494
	Age at baseline^2^	−0.0111	−0.0210	−0.0012	<0.05
	Sex	23.7066	18.9848	28.4284	<0.001
	Educational level	14.0986	10.9813	17.2159	<0.001
	Hypertension	−3.3516	−9.9322	3.2291	0.318
	Coronary artery disease	−6.8034	−15.9464	2.3396	0.145
	Diabetes Mellitus	−6.7221	−19.1115	5.6674	0.287
	Hypercholesterolemia	−3.1397	−11.5171	5.2377	0.462
	History of smoking	−0.2115	−5.2738	4.8508	0.935
	Problematic alcohol use	−5.5676	−13.9310	2.7957	0.192
Stroop colour word test	Intercept	3.8380	3.6405	4.0356	<0.001
*(Attention & Mental speed)*	Estimated total anaesthesia time at baseline	−0.0004	−0.0005	−0.0002	<0.001
	Time (follow-up)	0.0167	0.0035	0.0298	<0.05
	Total anaesthesia time at baseline × time (minutes)	0.0003	0.0002	0.0004	<0.001
	Age at baseline	−0.0112	−0.0185	−0.0039	<0.001
	Age at baseline^2^	0.0003	0.0002	0.0003	<0.001
	Sex	−0.0684	−0.1025	−0.0343	<0.001
	Educational level	−0.1106	−0.1328	−0.0883	<0.001
	Hypertension	0.0203	−0.0273	0.0679	0.403
	Coronary artery disease	0.0062	−0.0600	0.0725	0.853
	Diabetes Mellitus	0.1233	0.0338	0.2128	<0.01
	Hypercholesterolemia	−0.0320	−0.0926	0.0285	0.299
	History of smoking	0.0447	0.0081	0.0812	<0.05
	Problematic alcohol use	−0.0014	−0.0617	0.0588	0.963
Letter digit substitution test	Intercept	53.7097	48.7314	58.6879	<0.001
*Information processing speed)*	Estimated total anaesthesia time at baseline	0.0038	−0.0008	0.0085	0.105
	Time (follow-up)	−0.5404	−0.7992	−02.816	<0.001
	Total anaesthesia time at baseline × time (minutes)	−0.0037	−0.0060	−0.0014	<0.005
	Age at baseline	0.0333	−0.1504	0.2169	0.723
	Age at baseline^2^	−0.0046	−0.0064	−0.0028	<0.001
	Sex	1.0792	0.2143	1.9440	<0.05
	Educational level	3.8817	3.3267	4.4366	<0.001
	Hypertension	−1.4776	−2.6778	−0.2774	<0.005
	Coronary artery disease	−0.5703	−2.2265	1.0859	0.500
	Diabetes Mellitus	−3.3152	−5.5605	−1.0699	<0.005
	Hypercholesterolemia	1.6566	0.1228	3.1905	<0.05
	History of smoking	−1.4072	−23.337	−0.4807	<0.005
	Problematic alcohol use	−0.0732	−1.5971	1.4507	0.925

CST: speed in seconds; higher scores mean slower task completion. VVLT: number of correct recalls; lower score means lower test performance. Stroop: time in seconds; a higher score is slower task completion. LDST: number of correct substitutions; a lower score means lower test performance.

The full model incorporated both demographic and health-related variables. Performance on the CST (estimate 0.0001 (95% CI 0 to 0.0002) *P* < 0.05) (executive functioning), Stroop (estimate 0.0002 (95% CI 0.0001 to 0.0002) *P* < 0.001) (selective attention and mental speed), and LDST (estimate −0.0037 (95% CI −0.006 to 0.0014) *P* < 0.005) (information processing speed), which was negatively impacted during the follow-up period by an increased time under general anaesthesia at baseline. In the full model, age and educational level remained substantial factors explaining decreased performance on all outcome variables.

Furthermore, health-related factors, such as smoking, hypertension, and diabetes, substantially negatively affect cognitive development in related domains, namely executive function, selective attention, mental speed, and information processing speed.

**Fig. 1:**
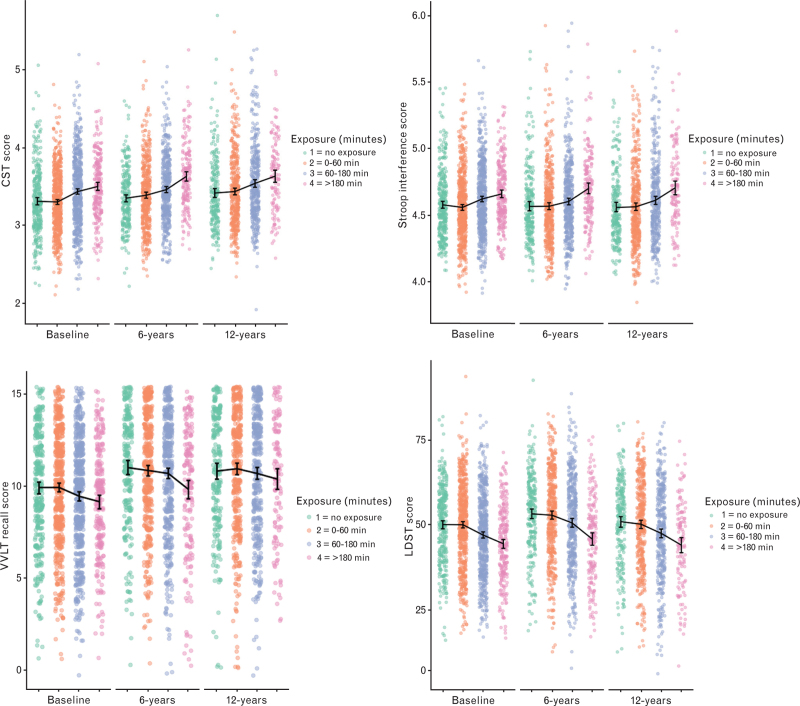
Scatterplots of outcome variables at the 12-year follow-up.

**Fig. 2:**
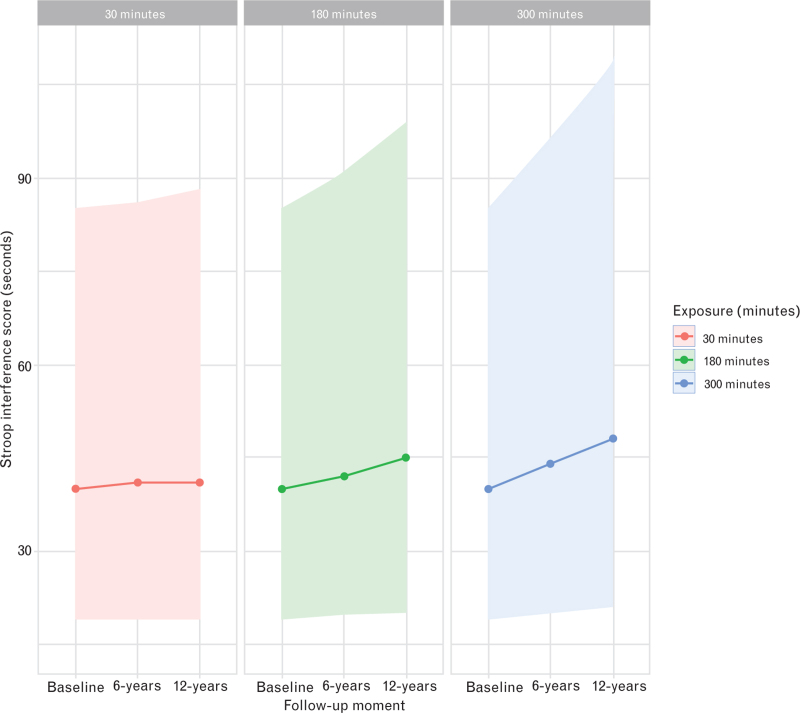
Using data from the basic mixed model, we calculated three theoretical cognitive trajectories, each with different exposure (measured in minutes) to surgery performed under general anaesthesia.

We have illustrated the impact of increased anaesthesia exposure in the two figures. Figure 1 presents boxplots of the primary outcome measures at the 12-year mark, categorised by the exposure level. Using our basic model, Fig. 2 plots three hypothetical cognitive trajectories from the Stroop Colour Word Test against varying baseline anaesthesia exposures. When comparing 30 min of baseline exposure to 180 min, the maximum calculated divergence over 12 years was 7 s.

CST: a higher score means slower task completion. VVLT: a lower score means lower test performance. Stroop: a higher score means slower task completion. LDST: a lower score means lower test performance.

We performed a post hoc analysis by adding the interaction term total time under general anaesthesia at baseline × age to check whether increased exposure to general anaesthesia at older ages would negatively affect cognitive development. However, there was no significant effect on the outcome variables.

## Discussion

The longitudinal assessment of 1823 participants within the MAAS cohort demonstrated a considerable negative impact of the total exposure time to surgical procedures under general anaesthesia on cognitive decline in three out of four tested domains: executive functioning, selective attention and mental speed, and information processing speed. These deleterious effects retained their significance when adjusted for recognised predictors of cognitive decline, indicating that increased exposure to procedures under general anaesthesia correlates with a decrease in cognitive functionality over an individual's lifespan. Although the effect seen in our model is robust, the overall discrepancy between theoretical individuals, for example, in the Stroop Color Word Test, does not exceed seven seconds.

### Comparison with previous studies

Various studies have investigated the longitudinal effects of anaesthesia and surgery on cognitive decline. In contrast to our results, Avidan *et al.* found no association between surgery or illness and changes in cognitive trajectory in a retrospective cohort of 575 patients.^25^ They used similar linear mixed-effects models and a comprehensive neurocognitive test battery to assess the cognitive function. However, data on the length of the surgical procedure were not included, and no correction for medical history was performed. Another prospective, multicentre cohort study also reported no association between surgery under general anaesthesia and cognitive impairment after major non-cardiac surgery or critical illness.^26^ This study focused on current hospital admissions without data on previous surgical exposure. In addition, the follow-up time of 12 months was relatively short compared with that in our study. Similar to our results, Schulte *et al.* reported a subtle decline in cognitive scores in a cohort of 1819 elderly adults who underwent surgery under general anaesthesia.^27^ In a large longitudinal study on cognitive functioning in middle-aged and elderly Danish twins, major surgery under general anaesthesia was associated with a minimally lower level of cognitive functioning. Preoperative cognitive functioning, underlying diseases, and lifestyle factors were more important for cognitive functioning in mid- and late life than surgery and anaesthesia.^28^ In addition, major surgery in the Whitehall II cohort was associated with a slight change in the long-term cognitive trajectories.^29^ This study investigated cognitive trajectories in a broader scope, including stroke and surgical and medical admissions. Medical admissions and stroke had a more significant effect on the decline in cognitive trajectories, indicating that the underlying disease could be of greater importance for lifetime cognitive decline.

Our results also suggest that accelerated cognitive decline during an individual's lifetime largely depends on the existence of systemic diseases rather than surgery and general anaesthesia alone. Previous results from the MAAS cohort support these findings regarding hypertension^24^ and diabetes mellitus.^30^ Similar evidence is available in patients with coronary artery disease. During a 6-year follow-up period of patients with coronary artery disease, all patients experienced a significant decrease in cognitive function compared to healthy individuals, independent of whether they underwent CABG surgery or nonsurgical management.^31^ Furthermore, a long-term decrease in cognitive functioning was found in patients 12 months and up to 7,5-years following coronary artery bypass surgery.^32,33^ One could argue that cardiovascular disease is the most likely reason for cardiovascular surgery and cognitive decline rather than vice versa. The same analogy can be made for the MAAS cohort. Participants with one or more comorbidities may be more prone to surgery under general anaesthesia. Despite adjusting our model for various covariates, distinguishing the impacts of the surgical procedure, anaesthesia, and comorbidities remains complex owing to their co-dependent interactions.

### Strengths and limitations of this study

The main strength of our study is its prospective nature combined with a long follow-up period. Previous studies on this subject were mainly retrospective and had shorter follow-up periods than those in the MAAS study. Furthermore, the cohort was stratified by design, and information was gathered through questionnaires and medical tests. Trained personnel use an extensive battery of neurocognitive tests. Thus, a broad range of cognitive domains have been extensively tested throughout the research period. Another strength is the use of linear mixed models, which can deal with missing data in a longitudinal design and allow for a better assessment of cognitive trajectories than previous statistical methods.^25,34^

Although the effect of total time under general anaesthesia is significant in several cognitive domains, assessing the relationship between anaesthesia exposure and cognitive decline presents several challenges and limitations in our study.

First, the potential effects of anaesthesia exposure on cognitive decline cannot be entirely separated from the broader effects of surgical events, including the systemic inflammatory response that accompanies surgery.^35^ While we focused on total time under anaesthesia, the intertwined effects of surgical stress and anaesthesia exposure are difficult to distinguish within a single patient. For example, minimally invasive procedures, which may extend the surgical duration, can reduce surgical stress and tissue damage, potentially balancing the cognitive effects of more prolonged anaesthesia exposure.

Second, the type and timing of surgical procedures were based on patient-reported data and recorded only at baseline. No data on intraoperative or postoperative complications were available, which may have influenced procedural duration and anaesthesia exposure. An experienced anaesthesiologist estimated the total time under anaesthesia based on reported procedures under normal circumstances. While this approach provides a reasonable estimate, it cannot account for intraoperative complications that could extend procedural time.

Additionally, historical data on procedural duration may not fully reflect modern perioperative practices. While some surgical procedures, such as robotic and laparoscopic surgeries, have become longer due to their complexity, many other procedures have become faster with the advent of enhanced recovery pathways and less invasive techniques.^36^ In the Netherlands, these advancements between 1991 and 2018 contributed to improved surgical outcomes, including reduced morbidity and mortality.^37,38^ However, this evolution complicates direct comparisons between the data from the MAAS cohort, where all surgeries before baseline occurred before 1993, and modern-day perioperative practices. The effects observed in the MAAS cohort could differ under current conditions due to these advancements.

Moreover, our study did not account for differences in anaesthesia types, such as the shift from volatile anaesthetics to total intravenous anaesthesia (TIVA). Emerging evidence suggests that TIVA may be associated with a lower incidence of postoperative cognitive dysfunction compared to volatile agents, potentially due to its anti-inflammatory and neuroprotective properties^39^ However, given the historical context of our cohort, where data were collected primarily in the 1990s and early 2000s, this factor was not assessed.

Lastly, while age remains a major contributor to cognitive decline, our analyses found that total time under general anaesthesia was still an independent, significant predictor of cognitive decline across several domains. Although its effect was smaller than age, anaesthesia exposure remains relevant. These findings highlight the multifactorial nature of cognitive decline, where age and anaesthesia exposure independently contribute to outcomes. Finally, the design of the MAAS cohort allowed us to study the long-term effects of the determinants of cognitive ageing. Due to this design, no information was available regarding short-term cognitive decline after surgery or possible correlations with long-term cognitive trajectories.

## Conclusion

Our results support the hypothesis that prolonged exposure to surgery under general anaesthesia may negatively affect long-term cognitive functioning. Although the effect is smaller than that of age, a significant reduction in executive functioning, selective attention, mental speed, and information processing speed was observed with increased anaesthesia exposure. This relatively small effect may still be clinically relevant to daily functioning. Lifestyle management and prevention remain crucial for promoting healthy cognitive ageing and could play a significant role in managing the ageing surgical population.

## Supplementary Material

Supplemental Digital Content
