# Supplementary material for: Anaesthesia as a risk factor for long-term cognitive decline: Results of the prospective MAAS cohort study
Source: Eur J Anaesthesiol. 2025 Feb 17;42(5):468–77. doi: 10.1097/EJA.0000000000002133 (PMC11972013; doi:10.1097/EJA.0000000000002133)
Supplement: Supplemental Digital Content [file ejanet-42-468-s001.docx]

**Appendix**

**Written linear mixed-effect models**

Basic model

1. Y_ti_ = ß_0_ + ß_1_⋅total time general anesthesia + ß_2_⋅time + ß_3_⋅age + ß_4_⋅age^2^ + ß_5_⋅sex + ß_6_⋅educational level + ß_6_⋅total time general anesthesia × time
   + e_ti_ + u_0i_ + u_1_⋅time

t = time points 1-2-3: baseline–6 years–12 years;
i = subject

Full model

1. Y_ti_ = ß_0_ + total time general anesthesia + ß_2_⋅time + ß_3_⋅age + ß_4_⋅age^2^ + ß_5_⋅sex + ß_6_⋅ education level + ß_7_⋅number of anesthesia sessions × time + ß_8_⋅hypertension + ß_9_⋅coronary artery disease + ß_10_⋅diabetes mellitus + ß_11_⋅hypercholesterolemia + ß_12_⋅history of smoking + ß_13_⋅alcohol abuse

+ e_ti_ + u_0i_ + u_1_⋅time

t = time points 1-2-3: baseline–6 years–12 years;
i = subject
